# The ORF1ab of Feline Coronavirus Plays a Critical Role in Regulating the Innate Immune Response

**DOI:** 10.3390/v17091282

**Published:** 2025-09-22

**Authors:** Haorong Gu, Chuqiao Xia, Hongtao Kang, Honglin Jia

**Affiliations:** State Key Laboratory for Animal Disease Control and Prevention, Harbin Veterinary Research Institute, Chinese Academy of Agricultural Sciences (CAAS), 678 Haping Street, Harbin 150069, China; guhaorong01@163.com (H.G.); 13083086377@163.com (C.X.)

**Keywords:** FCoVs, FECV, FIPV, ORF1ab, S protein, IFN-β

## Abstract

Feline coronaviruses (FCoVs) are divided into two groups: feline infectious peritonitis virus (FIPV) and feline enteric coronavirus (FECV). FIPV is responsible for the severe disease known as feline infectious peritonitis, while FECV typically causes mild symptoms, such as diarrhea, and often does not lead to any disease at all. Currently, it is not possible to distinguish between FIPV and FECV at the molecular level. Therefore, there is an urgent need to understand the molecular features of FIPV. Here, we generated a recombinant virus by replacing the ORF1ab region and the coding sequence for the spike (S) protein of an FECV with the corresponding sequences from FIPVs. The recombinant virus (recFECV-S_DF-2_-1ab_FIPV_) exhibited similar growth kinetics to its parental strain. Our analysis revealed that the replacement of the ORF1ab in the FECV caused significant alterations in protein expression within the host cells. Furthermore, the presence of the ORF1ab from the FIPV strain resulted in enhanced suppression of the innate immune response compared to the parental strain, as determined through proteomic and transcriptomic studies. Additionally, we demonstrated that the papain-like protease 2 (PL2^pro^) of the non-structural protein 3 (NSP3) from both FIPV and FECV functions in immune suppression, and the protease activity is required for this function.

## 1. Introduction

FCoV is an enveloped, single-stranded, positive-sense RNA virus belonging to the genus Coronavirus of the Coronaviridae [1]. The genome of FCoV is approximately 29 kilobases (kb) in length and contains seven open reading frames. These frames encode the polyprotein pp1ab (ORF1ab), which can be cleaved into 16 non-structural proteins (NSPs), structural proteins such as the spike protein (S), envelope protein (E), membrane protein (M), and nucleocapsid protein (N), and accessory proteins (ORF3 and ORF7). FCoVs can be categorized into two biotypes based on the clinical symptoms they cause: feline infectious peritonitis virus (FIPV) and feline enteric coronavirus (FECV). FECV often causes enteritis or is asymptomatic, while FIPV leads to feline infectious peritonitis (FIP), a severe and often fatal disease with a mortality rate reaching 100% without treatment. FCoVs are also classified into two genotypes: type I and type II. Type I FCoVs are widely distributed in nature, while type II FCoVs occur less frequently [2,3,4]. Epidemiological surveys have shown that type II arises from recombination between type I FCoVs and canine coronavirus (CCoV). Compared to type I FCoVs, type II FCoVs are easier to culture in vitro, which is why most research on FIP and FIPV focuses on type II FCoVs. Both genotypes can belong to either the FECV or FIPV biotype.

Although low-pathogenicity FCoV is highly contagious, only a small percentage of infected cats develop clinical FIP, and only a few FIP-affected cats shed FIPV [5]. Multiple studies have indicated that FIPV strains are more closely related to FCoVs found in fecal samples of cats living in the same environment but not to the FIPV isolates in other locations [2,4]. Phylogenetic analysis of FIPV strains from different cats in the same household reveals unique genetic signatures, suggesting independent emergence of the virus in each feline host [4]. Furthermore, oral inoculation of healthy cats with FIPV is not efficient in causing the development of FIP [6,7]. Therefore, the likelihood of horizontal transmission of FIPV is low, although it is possible [8,9]. The factors contributing to FIPV outbreaks in multi-cat environments are likely diverse [10,11,12].

The mechanism underlying the development and pathogenesis of FIPV is not clear. Like all coronaviruses, the FCoV genome shows high genetic variability. It has been proposed that mutations in the spike (S) gene, along with accessory genes 3c and 7b, may play a role in this process [13,14], but subsequent research has not fully validated these claims [15]. Recent studies, for instance, have demonstrated that inserting the S protein from virulent type II FIPV into the backbone of low-virulence type I FECV does not change the biotype of the virus [16]. Furthermore, recombinant virulent viruses with an S protein from a low-virulence type II virus continue to exhibit high virulence [16,17]. These findings suggest that the virulence of FIPV does not depend solely on the S protein. Instead, it is likely that a combination of multiple mutations plays a role in the pathogenesis of FIP. Some studies have suggested that antibody-dependent enhancement plays an important role in the development of FIP [18,19]. However, this phenomenon is not often observed under natural conditions [12].

The currently accepted theory on the pathogenesis of FIPV suggests that when cats are infected with FCoV, the accumulation of viral genetic mutations alters the target cells of the virus from intestinal epithelial cells to monocytes/macrophages, leading to FIP development. However, others argue that FECV and FIPV are two distinct types of FCoV that circulate independently within cat populations [20,21]. Previous studies have shown that highly virulent strains of certain viruses can replicate more effectively in monocytes and macrophages [22]. Additionally, the occurrence of FIP is associated with significant reductions in both natural killer cells and regulatory T cells, indicating that both innate immunity and adaptive immunity are being suppressed [23]. Recent studies indicate that the type II FIPV (DF2 strain) fails to induce the production of IFN β and suppresses the IFN β production induced by Sendai virus (SeV) or polyinosinic–polycytidylic acid (poly(I:C)) [24,25]. This suppression is closely related to the activity of non-structural protein NSP5, which disrupts the type I interferon signaling pathway by cleaving NEMO through its protease activity. In addition to NSP5, the NSP1 of FIPV plays a critical role in inhibiting the host immune response [24,26]. However, the immune suppression activities of FIPV are not claimed to be a unique feature that differentiates it from FECV.

In this study, we constructed a recombinant virus of recFECV-S_DF-2_-1ab_FIPV_, in which the ORF1ab region was replaced with the corresponding region from an FIPV. Our findings revealed that the ORF1ab of FIPV exhibits a stronger ability to suppress innate immunity. Additionally, we discovered that the PL2^pro^ of NSP3 has a strong inhibitory ability in interferon production, and this inhibitory effect is dependent on its deubiquitinase activity.

## 2. Materials and Methods

### 2.1. Cells, Viruses, and Antibodies

Crandell feline kidney (CRFK) cells were purchased from National Collection of Authenticated Cell Cultures (Shanghai, China; CAT#3101MAMGNO16). Felis catus whole fetus (Fcwf-4, CRL-2787) cells were obtained from ATCC. CRFK cells were maintained in Dulbecco’s modified Eagle’s medium (DMEM; Sigma Aldrich, Darmstadt, Germany; CAT#D6429-500ML) supplemented with 10% fetal bovine serum (FBS) (Clark Bioscience, Richmond, VA, USA; CAT#FB25015) and 100 U/mL penicillin, 100 μg/mL streptomycin (Gibco, Thermo Fisher Scientific, Waltham, MA, USA; CAT#15140-122), at 37 °C in an atmosphere of humidified air containing 5% (*v*/*v*) CO_2_. Fcwf-4 cells were maintained in Eagle’s minimum essential medium (EMEM, Wisent Biotechnology (Nanjing) Co., Ltd, Nanjing, Jiangsu, China; CAT#320-006-CL) supplemented with 10% FBS (Gibco, Thermo Fisher Scientific, Waltham, MA, USA; CAT#10099141C), 100 U/mL penicillin, 100 μg/mL streptomycina, and 1 mM Sodium Pyruvate (Gibco, Thermo Fisher Scientific, Waltham, MA, USA; CAT#11360-070) at 37 °C in an atmosphere of humidified air containing 5% (*v*/*v*) CO_2_. Feline bone marrow-derived macrophage cells (BMDMs) were maintained in RPMI-1640 medium (Gibco, Thermo Fisher Scientific (Suzhou) Instruments Co., Ltd., Suzhou, Jiangsu, China; CAT#C11875500BT) supplemented with 10% FBS (Gibco, Thermo Fisher Scientific, Waltham, MA, USA; CAT#10099141C) and 100 U/mL penicillin, 100 μg/mL streptomycin, at 37 °C in an atmosphere of humidified air containing 5% (*v*/*v*) CO_2_. The recFECV-S_DF-2_ was constructed in a previous study and preserved in this laboratory [27].

The mouse-derived polyclonal antibody against FIPV N protein was prepared in this experiment. Monoclonal Anti-β-Actin antibody (CAT#A5441-100UL) and DAPI (CAT#D9542-5MG) were purchased from Sigma Aldrich (Darmstadt, Germany). Goat anti-Mouse IgG (H+L) Alexa Fluor™ 488 (CAT#A11001) was purchased from Thermo Fisher Scientific (Waltham, MA, USA). IRDye^®^ 680RD Goat anti-Mouse IgG (H+L) (CAT#926-68070), IRDye^®^ 800CW Goat anti-Rabbit IgG (H+L) (CAT#926-32211), and IRDye^®^ 800CW Goat anti-Mouse IgG (H+L) (CAT#926-32210) were purchased from LI-COR Biosciences (Lincoln, NE, USA). Anti-HA Rabbit Polyclonal Antibody (CAT#51064-2-AP) was purchased from Proteintech (Wuhan, Hubei, China).

### 2.2. Rescue of the Recombinant Virus

The FIPV ORF1ab sequence was synthesized based on the NCBI database sequence (HLJ/HRB/2016/10, ACC#: KY566209.1) [28]. The recFECV-S_DF-2_-1ab_FIPV_ was constructed by a rapid reverse genetic system for feline coronavirus based on TAR cloning in yeast [27,29,30,31,32,33,34]. Two micrograms (μg) of infectious clone plasmid was added to 100 μL of DMEM and mixed thoroughly. Six μg of PEI was added to 100 μL of DMEM and mixed thoroughly. The plasmid mixture was combined with the PEI mixture and used to transfect the cells. After 6 h of transfection, the medium was replaced with DMEM containing 2% FBS.

### 2.3. Isolation and Culture of Feline BMDMs

Feline bone marrow-derived macrophages (BMDMs) were isolated and cultured following established protocols from previous studies [23,24,25]. In brief, a one-year-old, healthy cat was confirmed via PCR testing to be free from feline infectious viruses, including feline coronavirus, feline parvovirus, feline calicivirus, and feline herpes virus-1. To isolate the bone marrow, small openings were made at both ends of the femur and tibia of the cat using needles. Bone marrow cells were then repeatedly flushed using a 50 mL syringe. Following this, 40 mL of red blood cell (RBC) lysis buffer (Solarbio Science & Technology Co., Ltd., Beijing, China, CAT#R1010-500 mL) was added to lyse the red blood cells, and the cell pellet was resuspended in RPMI-1640 medium. The cell suspension was filtered (Falcon, Corning, NY, USA, CAT#352340) and further resuspended in RPMI-1640 medium supplemented with 10% FBS and 100 ng/mL granulocyte–macrophage colony-stimulating factor (GM-CSF) (Solarbio Science & Technology Co., Ltd., Beijing, China, CAT#P00130-50 µg). The cells were counted and seeded into a 24-well plate at a density of 1 × 10^6^ cells per well. Finally, the cells were cultured in an incubator at 37 °C with 5% CO_2_ for one week.

### 2.4. Viral Growth Kinetics

CRFK and BMDM cells were infected with the recFECV-S_DF-2_-1ab_FIPV_ at a multiplicity of infection (MOI) of 0.01. At 12 h, 24 h, 36 h, and 48 h postinfection, supernatant was collected and stored at −80 °C. The viral titer (TCID_50_) was determined in CRFK cells. The cells were seeded at a concentration of 5 × 10^5^ cells/mL. The viral suspension was diluted in a series of 10-fold dilutions (from 10^−1^ to 10^−6^), with six replicates for each dilution. Next, 100 µL of the diluted virus was added to each well, while 100 µL of DMEM was added to the control wells. After seven days of culture, cytopathic effects (CPEs) were observed and recorded for each dilution. The Reed–Muench method was then utilized to calculate the viral dilution required to infect 50% of the cells. Each viral suspension from the different time points was diluted independently three times, and the viral titer was evaluated in triplicate. The viral titer with three wells showing CPEs at the 10^−1^ dilution was considered to be 100 TCID50/mL. The limit of detection was 100 TCID_50_/_mL_.

### 2.5. Immunofluorescence Assay

The cells were washed three times with phosphate-buffered saline (PBS) after clear CPEs were observed, fixed at room temperature for 30 min, and then washed again and permeabilized using a 0.3% Triton X-100 solution for 5 min. They were blocked with 5% BSA in PBS buffer for 30 min before being stained with an anti-FCoV N antibody diluted to 1:1000 for 1 h. Following five more washes with PBS, the cells were incubated with anti-M Alexa 488 and DAPI, both diluted to 1:1000, for 1 h. The cells were washed five additional times with PBS and observed under a fluorescence microscope (Thermo Fisher Scientific, Waltham, MA, USA; EVOS FL Auto 2).

### 2.6. Electron Microscopy Identification

The rescued virus was inoculated into CRFK cells at an MOI of 0.1. After 72 h, the cells were broken through two freeze–thaw cycles and centrifuged at 60,000× *g* for 4 h at 4 °C. Following centrifugation, the pellet was resuspended in 8.75 mL of Hepes buffer (0.9% NaCl, 1 mM Hepes, pH 6.7) and kept in a refrigerator at 4 °C to dissolve. After 4 h, 625 µL of a 30% paraformaldehyde solution was added to achieve a final concentration of 2%. The virus was purified through centrifugation against a sucrose gradient (10%, 20%, and 30%) at 60,000× *g* for 4 h at 4 °C. After centrifugation, 38 mL of Hepes buffer was added and centrifuged again to purify the virus. The pellet was resuspended in 100 µL of Hepes buffer and placed in a refrigerator at 4 °C to dissolve overnight. The virus was then negatively stained and observed using electron microscopy.

### 2.7. Analysis of Gene Expression Using RT-qPCR

Total RNA was extracted using Trizol reagent (Takara Bio Inc., Beijing, China; CAT#9109). The reverse transcription process was completed by HiScript II Q Select RT SuperMix for qPCR (+gDNA wiper) (Vazyme Biotech Co., Ltd., Nanjing, Jiangsu, China; CAT#R233-01). Real-time RT-qPCR was performed using TB Green^®^ Premix Ex Taq™ II (Tli RNaseH Plus) (Takara Bio Inc., Beijing, China; CAT#RR820A) and the Fluorescent Quantitative PCR Instrument (Applied Biosystems, Thermo Fisher Scientific, Waltham, MA, USA; QuantStudio 5). The amplification procedure was as follows: 95 °C for 30 s, followed by 40 cycles of two steps (95 °C for 10 s and 60 °C for 30 s), 95 °C for 15 s, 60 °C for 1 min, and 95 °C for 15 s. The GAPDH gene served as the internal control. Three replicates were assayed for each individual transcript in each sample. Changes in gene expression relative to the control were calculated using the delta–delta cycle threshold (ΔΔCT) method. The primer pairs used were as follows: RIG-I F and R (TGTCGCCCTGGTTTAAGGAC; CCAAAAAGCCACGGAACCAG), MDA5 F and R (GGAGCTAACAGGCACCACTT; TGACCCGTGGCTACAAGAAC), and GAPDH F and R (GCCATCAATGACCCCTTCAT; GCCGTGGAATTTGCCGT). The primers for MDA5, RIG-I, and GAPDH have been validated through sequencing analysis of the amplicons. The primer pairs for IFN-β, ISG15, and IFIM1 were described previously [24]. All experiments were independently repeated three times.

### 2.8. Dual-Luciferase Reporter Assay

CRFK cells were plated in 24-well plates at 2.5 × 10^5^ per well and incubated until the cells reached about 80% confluency. Then, the cells were co-transfected with the reporter plasmid IFN-β-Luc [35], pRL-TK (Promega) encoding Renilla luciferase, entry vector (pCAGGs-3HA), or plasmids expressing the NSPs, using Lipofectamine 2000 regent (Invitogen, Thermo Fisher Scientific, Waltham, MA, USA; CAT#11668-019) transfection reagent. After 24 h, cells were stimulated with 20 hemagglutinating activity units of SeV. The luciferase activity was measured with a Dual-Luciferase^®^ Reporter Assay System (Promega Corporation, Madison, WI, USA; CAT#E1910). Successful transfection of the reporter plasmids was validated by measuring the luciferase activity of the cells stimulated with Sev. All experiments were independently repeated three times.

### 2.9. Transcriptomic Assay

CRFK cells were infected with the recombinant viruses at 0.1 MOI for 16 h. Three replicates were prepared for each group. Then, the cells were washed three times with pre-cooled PBS, and treated with 1 mL Trizol until the cells were fully lysed. Total RNA was extracted and subjected to the transcriptomic analysis (Shanghai Personal Biotechnology Co., Ltd., Shanghai, China).

Briefly, three μg total RNA was used for library preparation. Poly (A)-tailed mRNA was enriched, fragmented, and reverse-transcribed into cDNA. Purified double-stranded cDNA was repaired, adenylated, and ligated with adapters. Fragments with a size of 400–500 bp were selected, PCR-amplified, and purified using AMPure XP beads (Beckman Coulter, Beverly, CA, USA). The libraries were assessed using an Agilent 2100 Bioanalyzer, and then sequenced on the Illumina NovaSeq 6000 platform under PE150 mode.

The high-quality clean reads were aligned to the reference genome (https://ftp.ensembl.org/pub/release-110/fasta/felis_catus/dna/Felis_catus.Felis_catus_9.0.dna.toplevel.fa.gz, accessed on 4 May 2023) using HISAT2 (v2.1.0). The uniquely mapped paired-end reads were retained for downstream analysis. The read counts for each gene were quantified using HTSeq (v0.11.2). Differential gene expression analysis was performed using edgeR (v3.26.6). Briefly, read counts were first normalized using the TMM (Trimmed Mean of M-values) method to account for differences in sequencing depth. Gene-wise exact tests were then applied to calculate *p*-values for expression differences between groups. The resulting *p*-values were adjusted for multiple comparisons using the Benjamini–Hochberg (BH) method to control the false discovery rate (FDR). Significantly differentially expressed genes were defined as those with an FDR < 0.05, and fold change (FC) > 2 (up) or FC < 0.5 (down). A hypergeometric test was applied to identify GO terms and the Kyoto Encyclopedia of Genes and Genomes (KEGG) pathways that were significantly enriched among the DEGs compared to the whole genomic background. GO enrichment analysis was performed using topGO (v2.36.0), while KEGG pathway enrichment was conducted with KOBAS (v3.0.3).

### 2.10. Proteomic Assay

CRFK cells were infected with the recombinant viruses at 0.1 MOI for 20 h and rinsed three times with ice-cold PBS. The cells were collected by scraping and lysed by the SDT buffer (4% SDS, 100 mM Tris–HCl, pH 7.6). Three replicates were prepared for each group. The proteomic analysis was performed by Shanghai Applied Protein Technology Co., Ltd. (Shanghai, China).

Briefly, the lysates were boiled for 15 min. Following centrifugation at 14,000 g for 40 min, the supernatant was quantified using the BCA Protein Assay Kit. DTT was added to each sample to a final concentration of 40 mM and mixed at 600 rpm for 1.5 h at 37 °C. After cooling to room temperature, IAA was introduced at a final concentration of 20 mM to alkylate reduced cysteine residues, followed by a 30 min incubation in the dark. The samples were then transferred to 10 kDa Microcon filter units. Each sample was desalted using C18 cartridges (Empore™ SPE MCX, 30 μm, Waters, Milford, MA, USA), concentrated by vacuum centrifugation, and redissolved in 40 μL of 0.1% (*v*/*v*) formic acid. Peptide concentration was determined by measuring UV absorbance at 280 nm. For DIA analysis, indexed retention time (iRT) peptides were added to the sample. The peptides from each sample were analyzed by an Orbitrap TM Astral TM mass spectrometer (Thermo Fisher Scientific, Waltham, MA, USA) connected to a Vanquish Neo system liquid chromatograph (Thermo Fisher Scientific, Waltham, MA, USA) in the data-independent acquisition (DIA) mode.

DIA data were processed using DIA-NN (version 1.8.1) with the following parameters: trypsin as the enzyme, a maximum of one missed cleavage, and carbamidomethylation as a fixed modification. Oxidation and N-terminal acetylation were considered variable modifications. Protein identifications were filtered at a 1% FDR, which corresponds to 99% confidence. The resulting *p*-values (determined by a *t*-test) were adjusted for multiple comparisons using the BH method to control FDR. After annotation, significantly differentially expressed proteins were selected based on the following criteria: an FC greater than 1.5 (indicating upregulation) or less than 0.67 (indicating downregulation), with FDR less than 0.05. Additionally, presence–absence differential proteins were identified using a screening criterion where proteins were detected (non-empty values) in at least half of the replicates in one group, while being completely absent (empty values) in all replicates of the other group. These proteins were then searched against the KEGG database (https://www.genome.jp/kegg/, accessed on 28 October 2024) to obtain KEGG Orthology (KO) identifiers, which were subsequently mapped to KEGG pathways.

### 2.11. Western Blotting

CRFK cells were infected with the rescued virus at an MOI of 0.1, and lysed with lysis buffer 24 h later. After electrophoresis, the protein gel was transferred to a PVDF membrane. The membrane was incubated with primary antibodies—anti-FCoV-N and anti-β-actin—each diluted at a ratio of 1:3000 for 1 h. The secondary antibody, anti-M-800, was applied at a dilution of 1:10,000 for another hour. After washing with TBST, imaging was performed using an Infrared Imaging System (LI-COR Biosciences, Lincoln, NE, USA; Odyssey CLX).

For the Western blotting of PL2^pro^ and its mutants, cell lysates from double-luciferase reporter gene assays were used. The PL2^pro^ and its mutants were detected with anti-HA. β-actin was used as an internal control.

### 2.12. Plasmid Construction

The coding sequences of the NSPs were amplified and cloned into the *Sph*I site of the pCAGGS-3HA vector, placing a 3 × HA tag at the N-terminus of the protein. The PL1^pro^ domain corresponds to amino acids (aa) 199–462 of NSP3, while the PL2^pro^ domain spans aa 614–899. Using a similar method, the plasmids for the PL2^pro^ mutants (C99A, H252A, and D265A, numbered from the first aa of PL2^pro^) were generated. The RIG-I and MDA5 genes were amplified from the genomic DNA of CRFK cells and subsequently cloned into the *Sph*I site of the pCAGGS-3Flag vector, which also places a 3 × Flag tag at the N-terminus of the protein.

## 3. Results

### 3.1. Rescue of the recFECV-S_DF-2_-1ab_FIPV_

**Figure 1:**
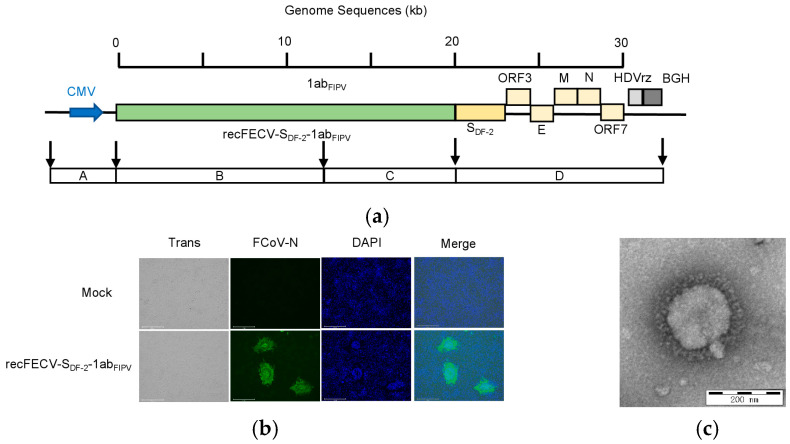
Construction and in vitro characterization of recFECV-S_DF-2_-1ab_FIPV_. (**a**) Schematic diagram of recFECV-S_DF-2_-1ab_FIPV_ cDNA clone. The full-length FECV-S_DF-2_-1ab_FIPV_ genome was assembled from four fragments. The A fragment contains a CMV promoter. The D fragment contains not only the S protein from DF-2 and the following part of the FECV genome but also the HDV ribozyme and the BGH transcription signal sequence. (**b**) IFA confirmed the successful rescue of the virus recFECV-S_DF-2_-1ab_FIPV_. (**c**) Electron microscopy images showed recFECV-S_DF-2_-1ab_FIPV_ particles purified from the supernatant of infected cells.

In the current study, we constructed a recombinant virus, in which the entire ORF1ab of the recFECV-S_DF-2_ [27] was replaced with the corresponding sequence from a type I FIPV strain [28] (Figure 1a). The full length of the infectious clone was sequenced using the second-generation whole-plasmid sequencing method by Sangon Biotech (Shanghai) Co., Ltd. (Shanghai, China). The results indicated that no mutations occurred in the viral genomic sequence (Supplementary Material: sequence 1).

The recombinant virus (recFECV-S_DF-2_-1ab_FIPV_) was rescued by transfecting CRFK cells with the plasmid containing the infectious clone. The expression of structural proteins was confirmed by staining the capsid protein N using an immunofluorescence assay (IFA) (Figure 1b). Three days after transfection, clear CPEs formed by the rescued virus were found in the transfected cells, while untransfected cells showed no CPEs. Virus particles exhibiting typical spike structures were observed under a transmission electron microscope (Figure 1c). These results indicate that we have successfully rescued the recFECV-S_DF-2_-1ab_FIPV_.

### 3.2. Growth Kinetics of the recFECV-S_DF-2_-1ab_FIPV_

**Figure 2:**
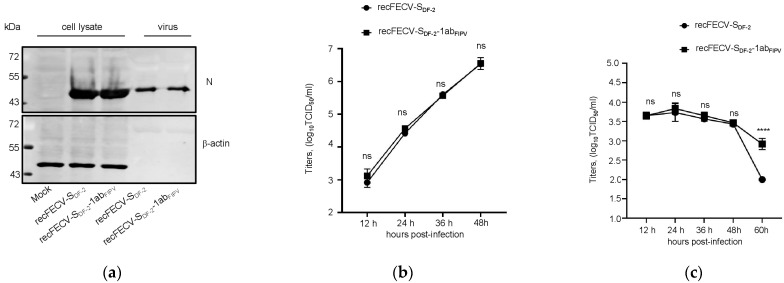
Construction and in vitro characterization of recFECV-S_DF-2_-1ab_FIPV_. (**a**) CRFK cells were infected with recFECV-S_DF-2_-1ab_FIPV_ and recFECV-S_DF-2_ at an MOI of 0.01. The expression of N protein was detected after 24 h post viral infection. (**b**) Growth curves of recFECV-S_DF-2_-1ab_FIPV_ and recFECV-S_DF-2_ in CRFK cells with an original MOI of 0.01. (**c**) Growth curves of recFECV-S_DF-2_-1ab_FIPV_ and recFECV-S_DF-2_ in BMDM cells with an original MOI of 0.01. The data shown represent the means ± SD, and all experiments were repeated three times. Asterisks indicate statistical significance as determined by two-way ANOVA; **** *p* < 0.0001.

We examined the growth kinetics of recFECV-S_DF-2_-1ab_FIPV_ in CRFK cells to investigate whether replacing the ORF1ab would affect the replication ability of the virus. Initially, we observed no differences between the recFECV-S_DF-2_-1ab_FIPV_ and the parental strain up to 48 h (Figure 2a,b). To further assess the replication rates of recFECV-S_DF-2_-1ab_FIPV_ in feline macrophages, we isolated monocytes from feline bone marrow and stimulated them in vitro with GM-CSF (100 ng/mL) for one week to induce differentiation into mature macrophages. We then infected these macrophages with each strain of virus at an MOI of 0.01 and measured the viral titers in the cell supernatants. The results indicated that the virus only replicated slightly, and there was still no significant difference between recFECV-S_DF-2_-1ab_FIPV_ and recFECV-S_DF-2_ during the first 48 h. (Figure 2c). However, at 60 h, the viral titer of recFECV-S_DF-2_ significantly decreased, while the titer of recFECV-S_DF-2_-1ab_FIPV_ remained relatively high. These findings suggest that the ORF1ab may play a role in virus burden during the later stages of infection.

### 3.3. Infection of recFECV-S_DF-2_-1ab_FIPV_ Causes Profound Changes in Protein Expression in CRFK Cells

**Figure 3:**
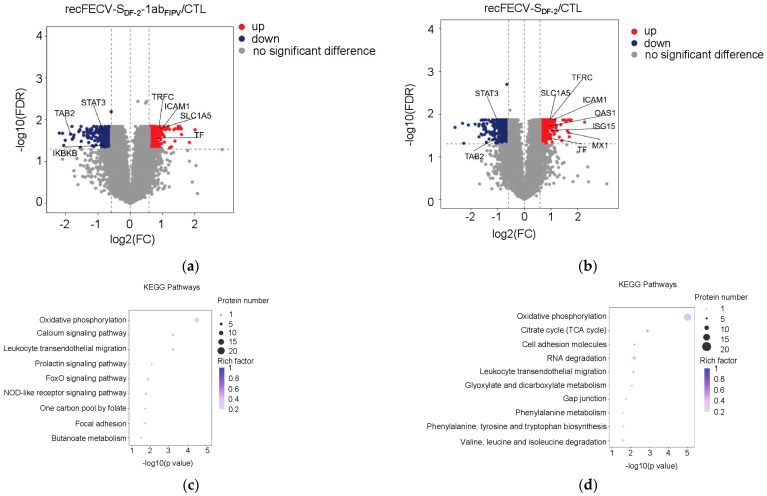
Changes in protein expression in CRFK cells infected with the recFECV-S_DF-2_-1ab_FIPV_ (**a**) and the recFECV-S_DF-2_ (**b**). X-axis indicates log2FC, and Y-axis indicates −log10 (FDR). Up- and downregulated genes were filtered based on statistical significance (FDR < 0.05, FC > 1.5 for upregulation or FC < 0.67 for downregulation). (**c**,**d**) KEGG enrichment of differentially expressed proteins in the cells infected with recFECV-S_DF-2_-1ab_FIPV_ (**c**) and recFECV-S_DF-2_ (**d**). The x-axis is the −log10 (FDR), and the y-axis is the name of each pathway entry. The size of the dot indicates the number of genes enriched, and the depth of the color indicates the size of the enrichment factor.

To investigate the alterations in protein expression caused by the recFECV-S_DF-2_-1ab_FIPV_ infection, we conducted a proteomic analysis of CRFK cells infected with the virus at an MOI of 0.1 for 20 h. The analysis identified a total of 796 differentially expressed proteins in the infected cells compared to the uninfected controls. Among these, 490 proteins were upregulated and 306 were downregulated, both showing statistical significance (FDR < 0.05, FC > 1.5 for upregulation or FC < 0.67 for downregulation) (Figure 3a; Supplementary Table S1). For the CRFK cells infected with recFECV-S_DF-2_, we identified 1197 differentially expressed proteins compared to the uninfected cells, with 664 significantly upregulated and 533 downregulated (FDR < 0.05, FC > 1.5 for upregulation or FC < 0.67 for downregulation) (Figure 3b; Supplementary Table S2). The KEGG pathway enrichment analysis revealed different profiles of altered protein expression in the cells infected with recFECV-S_DF-2_-1ab_FIPV_ and recFECV-S_DF-2_ (Figure 3c,d), except for the oxidative phosphorylation and leukocyte transendothelial migration pathway. Notably, several interferon-stimulated genes (ISGs) were absent from the list of differentially expressed genes in cells infected with recFECV-S_DF-2_-1ab_FIPV_ (FDR < 0.05).

### 3.4. The Presence of ORF1ab of FIPV in the Recombinant Virus Enhances Innate Immune Suppression

**Figure 4:**
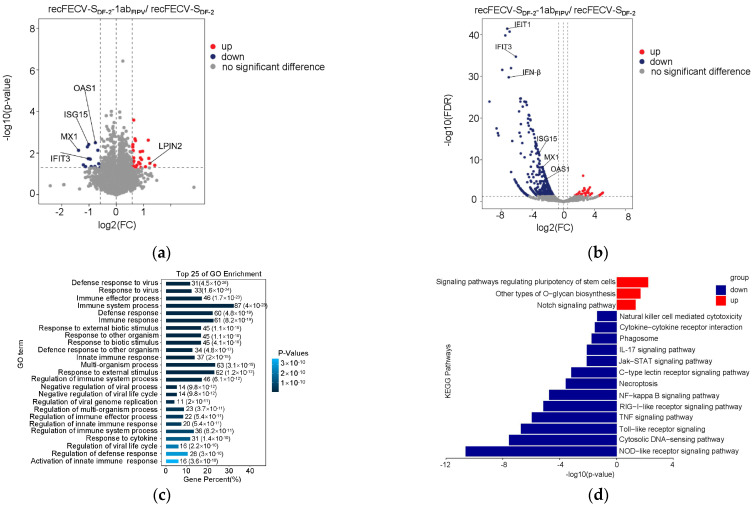
The ORF1ab of FIPV exhibits enhanced activity of innate immune suppression. (**a**) Comparison of the protein expression in the cells infected with the recFECV-S_DF-2_-1ab_FIPV_ and the recFECV-S_DF-2_. X-axis indicates log2 FC, and Y-axis indicates −log10 (*p*-value). Up- and downregulated genes were filtered based on statistical significance (*p* < 0.05, FC > 1.5 for upregulation or FC < 0.67 for downregulation). (**b**) Comparison of the gene transcription in the Fcwf-4 cells infected with the recFECV-S_DF-2_-1ab_FIPV_ and the recFECV-S_DF-2_. X-axis indicates log2 FC, and Y-axis indicates −log10 (FDR). Up- and downregulated genes were filtered based on statistical significance (FDR < 0.05, FC > 2 for upregulation or FC < 0.5 for downregulation). (**c**) Top25 in GO enrichment for differentially expressed genes. The color from dark to light represents high to low q-values, and the x-axis of the bar graph represents the proportion of differentially expressed genes enriched in each term relative to the total number of associated genes, with the number next to each bar representing the number of differentially expressed genes in each term. (**d**) Enrichment of selected KEGG pathways for differentially expressed genes. The X-axis indicates −log10 (*p*-value), and the y-axis is the name of each pathway entry. The pathways involved in upregulated proteins are indicated in red, and those involved in downregulated proteins are indicated in blue.

We compared the protein expression profiles in the cells infected with recFECV-S_DF-2_-1ab_FIPV_ to those in cells infected with its parental strain. We noted the overexpression of certain ISGs in cells infected with recFECV-S_DF-2_; this was not observed in cells infected with recFECV-S_DF-2_-1ab_FIPV_. Most of the differentially expressed proteins we identified were filtered out after applying multiple testing correction to the *p*-values, likely due to the relatively low number of differentially expressed proteins. As a result, *p*-values were not corrected for this comparison. The results revealed a total of 43 proteins that were differentially expressed, with 27 exhibiting increased expression and 16 showing decreased expression (*p* < 0.05, FC > 1.5 for upregulation or FC < 0.67 for downregulation) (Figure 4a; Supplementary Table S3). As noted, several ISGs displayed lower expression levels in cells infected with recFECV-S_DF-2_-1ab_FIPV_. To investigate whether similar differences in innate immune suppression could be observed in macrophages, we infected a macrophage-like cell line, Fcwf-4, at a dose of 0.1 MOI for 16 h and performed a transcriptomic analysis. The six transcriptome samples generated paired reads ranging from 26, 046, and 534 to 31, 785, and 660, with an average of 28, 614, and 127 reads per sample (Supplementary Table S4). The Q30 base percentage was greater than 99% for all samples. When comparing the cells infected with recFECV-S_DF-2_ to those infected with recFECV-S_DF-2_-1ab_FIPV_, a total of 578 genes exhibited differential expression. Among these, 47 genes were more highly expressed, while 531 genes showed reduced expression in the cells infected with recFECV-S_DF-2_-1ab_FIPV_ (FDR < 0.05, FC > 2 for upregulation or FC < 0.5 for downregulation). Notable examples of these genes include IFN-β and several interferon-stimulated genes (ISGs) such as IFIT-1, IFIT-3, ISG15, MX1, and OAS1 (Figure 4b; Supplementary Table S5). Gene Ontology (GO) analysis indicated that immune-related terms were significantly enriched among the genes differentially expressed in cells infected with recFECV-S_DF-2_-1ab_FIPV_ (Figure 4c). Additionally, KEGG enrichment analysis demonstrated that innate immune response pathways, including the NF-κB, RIG-I-like receptor, and NOD-like receptor signaling pathways, were significantly suppressed in these cells (Figure 4d). These findings suggest that the ORF1ab of this strain of FIPV has an enhanced immune suppression effect in macrophages as well.

### 3.5. NSP1 and NSP3 Are Involved in the Function of Innate Immune Suppression

**Figure 5:**
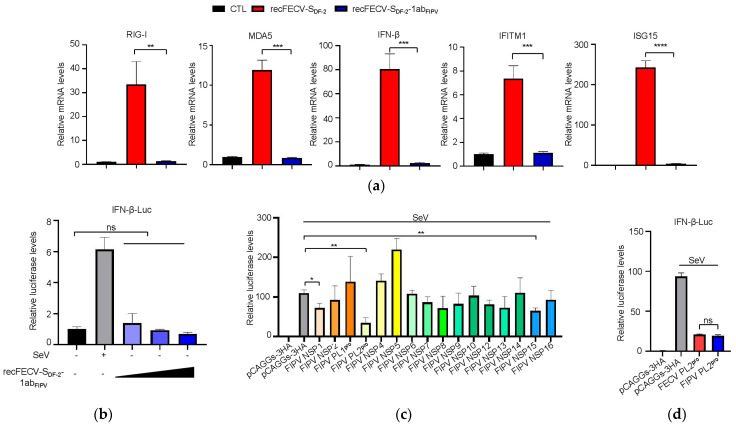
NSP1 and NSP3 are involved in the function of innate immune suppression. (**a**) RT PCR analysis of the transcription of the selected genes (RIG-I, MDA5, IFN-β, ISG15, and IFITM1) in recFECV-S_DF-2_-1ab_FIPV_-infected, recFECV-S_DF-2_-infected, and uninfected Fcwf-4 cells. Fcwf-4 cells (5 × 10^5^/well) were infected with 0.1 MOI recFECV-S_DF-2_ or recFECV-S_DF-2_-1ab_FIPV_. After 24 h, cells were collected and subjected to the analysis. The uninfected Fcwf-4 cells served as a negative control, while the GAPDH gene acted as an internal control. (**b**) CRFK cells (5 × 10^5^/well) were transfected with 400 ng/well of the IFN-β-Luc plasmid and 40 ng/well of the pRL-TK plasmid. At 24 h post-transfection, cells were stimulated by SeV or 0.01, 0.1, or 1 MOI of recFECV-S_DF-2_-1ab_FIPV_. The relative luciferase activity was determined after 20 h. The uninfected CRFK cells served as the negative control, while the CRFK cells infected with SeV acted as the positive control. (**c**) CRFK cells were transfected with the IFN-β-Luc and pRL-TK plasmids, together with 500 ng/well of the empty vector or the plasmid expressing each NSP. At 24 h post-transfection, cells were stimulated by SeV. The positive and negative controls were established as described in b. (**d**) CRFK cells were transfected with the IFN-β-Luc and pRL-TK plasmids, together with 500 ng/well of the empty vector or the plasmids expressing the PL2^pro^ of FECV or FIPV. The positive and negative controls were established as described in b. Differences between two experimental groups were analyzed using Student’s *t*-test. The data shown represent the means ± SD (*n* = 3), and all experiments were repeated three times. The significant differences are indicated as follows: * *p* < 0.05, ** *p* < 0.01, *** *p* < 0.001, and **** *p* < 0.0001.

To confirm the immune suppression effect of recFECV-S_DF-2_-1ab_FIPV_, as indicated by our proteomic and transcriptomic analyses, we measured the mRNA levels of RIG-I, MDA5, IFN-β, and ISGs (ISG15 and IFITM1) in the cells infected with the virus using quantitative reverse transcription PCR (RT-qPCR) in cells infected with either recFECV-S_DF-2_-1ab_FIPV_ or the parental strain virus, recFECV-S_DF-2_. As expected, we observed upregulation in the expression of all these genes solely in the cells infected with the parental strain (recFECV-S_DF-2_), while no clear increase was seen in the cells infected with the recFECV-S_DF-2_-1ab_FIPV_ (Figure 5a). Next, we examined the innate immune suppression effect of recFECV-S_DF-2_-1ab_FIPV_ infection in CRFK cells transfected with an IFN-Luc reporter plasmid. The results indicated that IFN-β expression was successfully stimulated in the cells infected with SeV. However, recFECV-S_DF-2_-1ab_FIPV_ failed to stimulate IFN-β expression (Figure 5b).

The ORF1ab encodes 16 NSPs. NSP1 and NSP5 derived from FIPVs have been reported to inhibit innate immune responses [24,26]. To determine whether these NSPs contribute to the innate immune suppression effects, we analyzed the effect of ectopic expression of each NSP on IFN expression induced by SeV in CRFK cells. We co-transfected CRFK cells with HA-tagged NSPs and the IFN-Luc reporter plasmid. SeV was introduced to the cells 24 h later to stimulate expression of the IFN-β promoter. As illustrated in Figure 5c, we identified two NSPs (NSP1 and NSP3) of this FIPV strain that exhibited an inhibitory effect on IFN-β expression. We compared the inhibitory activity of NSP3 from FIPV with that of NSP3 from FECV. Our findings indicated that NSP3 demonstrated no difference in innate immune suppression between FIPV and FECV.

### 3.6. The PL2 Protease Activity of NSP3 Is Required for Its Role in Innate Immune Suppression

**Figure 6:**
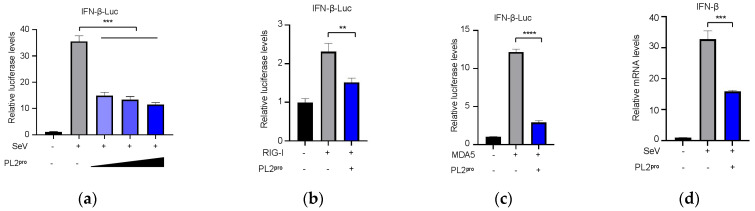

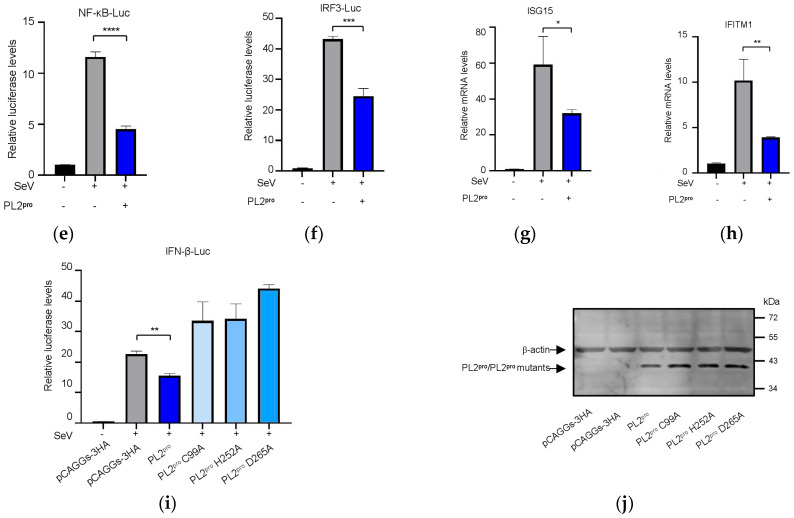
The enzymatic activity of PL2 protease is required for its role in innate immune suppression. (**a**). CRFK cells were co-transfected with 400 ng/well of IFN-β-Luc plasmid and 10 ng/well of pRL-TK plasmid, along with 400 ng/well of pCAGGs-3HA or increasing amounts of FIPV-PL2^pro^ (100 ng/well, 200/well, 400 ng/well). At 24 h, the cells were infected with SeV for 20 h. The cells were collected and the luciferase activities were measured. Cells transfected with pCAGGs-3HA served as the negative control, while those transfected with the pCAGGs-3HA and subsequently infected with SeV were used as the positive control. (**b**,**c**). CRFK cells were co-transfected with 400 ng/well of pCAGGs-3Flag, pCAGGs-3Flag-RIG-I (**b**), or pCAGGs-3Flag-MDA5 (**c**) and 400 ng/well of the reporter plasmid of IFN-β promoter and 10 ng/well of pRL-TK, along with 400 ng/well of pCAGGs-3HA or FIPV-PL2^pro^. At 24 h, the cells were collected and the luciferase activities were measured. Cells transfected with pCAGGs-3HA and pCAGGs-3Flag served as the negative control, while those transfected with pCAGGs-3HA and pCAGGs-3Flag-RIG-I (**b**) or pCAGGs-3Flag-MDA5 (**c**) were used as the positive control. (**d**). CRFK cells were transfected with 400 ng/well pCAGGs-3HA and FIPV-PL2^pro^ for 24 h and then infected with SeV for 20 h. The cells were collected and the mRNA levels of IFN-β were evaluated by RT-qPCR. The GAPDH gene served as an internal control. (**e**,**f**). CRFK cells were co-transfected with 400 ng/well of a reporter plasmid of the NF-κB promoter (**e**) or IRF3 promoter (**f**), 40 ng/well of pRL-TK, and 400 ng/well of pCAGGs-3HA or FIPV-PL2^pro^. At 24 h, the cells were infected with SeV. (**g**,**h**). CRFK cells were transfected with FIPV-PL2^pro^ (400 ng) for 24 h and then infected with SeV for 20 h, and the mRNA levels of ISG15 (**g**) and IFITM1 (**h**) were evaluated by RT-qPCR. The GAPDH gene served as an internal control. (**i**). CRFK cells were co-transfected with 400 ng/well of the IFN-β reporter plasmid and 10 ng/well of pRL-TK, along with 400 ng/well of pCAGGs-3HA, FIPV-PL2^pro^, PL2^pro^ C99A, PL2^pro^ H252A, or PL2^pro^ D265A for 24 h, and then infected with SeV for 20 h. (**j**). The expression of PL2^pro^ or the mutants was detected using the cell lysate in (**i**). The β-actin protein was used as an internal control. Differences between two experimental groups were analyzed using Student’s *t*-test. The data shown represent the means ± SD (*n* = 3), and all experiments were repeated three times. The significant differences are indicated as follows: * *p* < 0.05, ** *p* < 0.01, *** *p* < 0.001, and **** *p* < 0.0001.

To confirm the inhibitory role of PL2^pro^ of NSP3 in IFN-β production, CRFK cells were transfected with the IFN-β-Luc reporter plasmid along with varying amounts of plasmid expressing PL2^pro^. Luciferase activity was measured after stimulation with SeV, RIG-I, or MDA5. As shown in Figure 6a–c, activation of the IFN-β promotor could be inhibited by expressing PL2^pro^. The inhibitory effect of the PL2^pro^ on the production of IFN-β mRNA was confirmed by RT-qPCR (Figure 6d). To identify which transcription factor was inhibited by PL2^pro^, we co-transfected CRFK cells with the NF-κB-Luc or IRF3-Luc reporter plasmids and PL2^pro^ and stimulated them with SeV. The results indicated that PL2^pro^ significantly reduced the activation of both NF-κB and IRF3 in mediating IFN production (Figure 6e,f). Furthermore, the inhibitory effect of PL2^pro^ on the production of ISG15 and IFITM1 mRNA could be observed through the RT-qPCR (Figure 6g,h). We then constructed plasmids expressing PL2^pro^ with mutated enzymatic sites to evaluate their effect on IFN-β production. The results showed that the inhibitory effect of PL2^pro^ on IFN-β production was dependent on its protease activity (Figure 6i). The results of Western blotting showed that the mutants of PL2^pro^ could be stably expressed (Figure 6j).

## 4. Discussion

In feline populations, FECV is highly prevalent, primarily causing enteritis in juvenile cats while often remaining asymptomatic in adult cats. In contrast, FIPV occurs sporadically but can cause severe disease. Currently, the development of vaccines for FIP faces several challenges. Firstly, different serotype strains do not offer cross-protection, which limits the clinical efficacy of vaccines. Additionally, antibody-dependent enhancement (ADE) induced by the vaccines could worsen the disease [36]. Clinically, cases of FIP can be categorized as either effusive (wet) or non-effusive (dry). Effusive cases are characterized by the presence of ascites, pleural effusion, and pericardial effusion, while non-effusive cases often present with multiple pyogranulomas. Mixed forms can also occur. In the case of diagnostics, although immunohistochemical staining (IHC) for detecting the virus in infected tissues is considered an effective method for laboratory confirmation of FIP [37], this approach does not enable in vivo detection, particularly in cases where ascites is not produced, making clinical diagnosis more challenging. Therefore, it is urgent to determine the genetic features of FIPV, which will favor the development of molecular diagnostic tools.

In this study, we found that the presence of the ORF1ab derived from an FIPV strain enhanced the immune suppression activity of the virus in both CRFK and Fcwf-4 cells. This finding provides new insights into the biological features of FIPV. A previous study revealed that interferon production is differentially induced when Fcwf-4 cells are infected with various virus strains. In that study, the virulent strains (79-1146, identical to DF-2 and UCD-1) exhibited stronger inhibitory effects on interferon expression, although an avirulent strain (79-1683) demonstrated a similar capability [25]. It would be interesting to investigate whether the enhanced immune suppression caused by ORF1ab is a general feature of FIPVs and whether it occurs in vivo.

In addition, it has been suggested that mutations in the FECV genome lead to biotype conversion of the virus [2,4]. However, this hypothesis remains unverified, partly due to a lack of understanding of the distinct biological characteristics of FIPV and because studies on the pathogenesis of the virus largely rely on animal challenge experiments. A previous study reported that a chimeric type I FCoV containing sequences derived from type II FIPV, including ORF1b and S, 3a, 3b, and 3c, failed to induce clinical signs of FIP, indicating that these sequences are not sufficient to enhance pathogenicity [38]. This suggests that other sequences may play a role in defining the distinct biological characteristics of the viruses. It would be more interesting to investigate whether the enhanced suppression of the innate immune response by ORF1ab of FIPV is related to the pathogenesis of the virus through animal challenge experiments.

In previous studies, NSP1 and NSP5 of FIPV were shown to play important roles in evading the innate immune response [24,26]. In our current study, we also found that NSP1 exhibits an inhibitory effect on the innate immune response. However, NSP5 derived from the current strain of FIPV did not demonstrate any inhibitory function. Instead, we identified that PL2^pro^ fulfills the role of inhibiting IFN-β production. These findings suggest that the inhibitory effects may be strain-specific. Our data revealed that expression of PL2^pro^ could inhibit the activation of the IFN-β promoter stimulated by overexpression of RIG-1 or MDA5, which indicated that the target protein of PL2^pro^ might exist downstream of the RNA sensors. However, PL2^pro^ in other coronaviruses has been reported to have multiple targets for mediating innate immune evasion [39,40]. Therefore, multiple mechanisms might exist for the role of PL2^pro^ in regulating the innate immune response. Unfortunately, our data showed that the PL2^pro^ from both FIPV and FECV functions in immune suppression. Thus, detailed mapping within the ORF1ab using recombinant viruses with exchanged fragments of FIPV and FECV is still necessary for further investigation to identify the core factors associated with ORF1ab that contribute to this immune suppression role.

In summary, we have successfully constructed a recombinant virus harboring the ORF1ab from a virulent FIPV strain. Our findings suggest that ORF1ab from FIPV possesses a unique feature related to immune suppression. It remains unclear why ORF1ab exhibits an enhanced inhibitory function in innate immune responses, especially since PL2^pro^ from FECV and FIPV exhibits similar inhibitory effects. Therefore, additional investigation is needed to address this issue.

## Data Availability

All data are available in the manuscript.

## Supplementary Materials

The following supporting information can be downloaded at: https://www.mdpi.com/article/10.3390/v17091282/s1, Table S1: Differentially expressed proteins between cells infected with recFECV-S_DF-2_-1ab_FIPV_ and CTL; Table S2: Differentially expressed proteins between cells infected with recFECV-S_DF-2_ and CTL; Table S3: Differentially expressed proteins between cells infected with recFECV-S_DF-2_-1ab_FIPV_ and recFECV-S_DF-2_; Table S4: The paired-end reads of cells infected with recFECV-S_DF-2_-1ab_FIPV_ and recFECV-S_DF-2_; Table S5: Differentially expressed genes between cells infected with recFECV-S_DF-2_-1ab_FIPV_ and recFECV-S_DF-2_.

## Abbreviations

The following abbreviations are used in this manuscript:

FIP	Feline infectious peritonitis
FIPV	Feline infectious peritonitis virus
FECV	Feline enteric coronavirus
FCoV	Feline coronavirus
IFN-β	Interferon β
S	Spike protein
E	Envelope protein
M	Membrane protein
N	Nucleocapsid protein
PL2	Papain-like protease 2
CRFK	Crandell feline kidney
Fcwf-4	Felis catus whole fetus-4
Luc	Luciferase
SeV	Sendai virus
NSPs	Non-structural proteins
DMEM	Dulbecco’s modified Eagle’s medium
PBS	Phosphate-buffered saline
CPE	Cytopathic effects
GO	Gene Ontology
KEGG	Kyoto Encyclopedia of Genes and Genomes
ISGs	Interferon-stimulated genes
FDR	False discovery rate
EMEM	Eagle’s minimum essential medium
RT-qPCR	Quantitative reverse transcription PCR
